# Isolation and detection of DNA–protein crosslinks in mammalian cells

**DOI:** 10.1093/nar/gkad1178

**Published:** 2023-12-12

**Authors:** Ignacio Torrecilla, Annamaria Ruggiano, Kostantin Kiianitsa, Ftoon Aljarbou, Pauline Lascaux, Gwendoline Hoslett, Wei Song, Nancy Maizels, Kristijan Ramadan

**Affiliations:** The MRC Weatherall Institute of Molecular Medicine, Department of Oncology, John Radcliffe Hospital, University of Oxford, Oxford, OX3 9DS, UK; The MRC Weatherall Institute of Molecular Medicine, Department of Oncology, John Radcliffe Hospital, University of Oxford, Oxford, OX3 9DS, UK; Department of Immunology, University of Washington, Seattle, WA 98195-7350, USA; The MRC Weatherall Institute of Molecular Medicine, Department of Oncology, John Radcliffe Hospital, University of Oxford, Oxford, OX3 9DS, UK; The MRC Weatherall Institute of Molecular Medicine, Department of Oncology, John Radcliffe Hospital, University of Oxford, Oxford, OX3 9DS, UK; The MRC Weatherall Institute of Molecular Medicine, Department of Oncology, John Radcliffe Hospital, University of Oxford, Oxford, OX3 9DS, UK; The MRC Weatherall Institute of Molecular Medicine, Department of Oncology, John Radcliffe Hospital, University of Oxford, Oxford, OX3 9DS, UK; Department of Immunology, University of Washington, Seattle, WA 98195-7350, USA; Department of Biochemistry, University of Washington, Seattle, WA 98195-7350, USA; The MRC Weatherall Institute of Molecular Medicine, Department of Oncology, John Radcliffe Hospital, University of Oxford, Oxford, OX3 9DS, UK

## Abstract

DNA–protein crosslinks (DPCs) are toxic DNA lesions wherein a protein is covalently attached to DNA. If not rapidly repaired, DPCs create obstacles that disturb DNA replication, transcription and DNA damage repair, ultimately leading to genome instability. The persistence of DPCs is associated with premature ageing, cancer and neurodegeneration. In mammalian cells, the repair of DPCs mainly relies on the proteolytic activities of SPRTN and the 26S proteasome, complemented by other enzymes including TDP1/2 and the MRN complex, and many of the activities involved are essential, restricting genetic approaches. For many years, the study of DPC repair in mammalian cells was hindered by the lack of standardised assays, most notably assays that reliably quantified the proteins or proteolytic fragments covalently bound to DNA. Recent interest in the field has spurred the development of several biochemical methods for DPC analysis. Here, we critically analyse the latest techniques for DPC isolation and the benefits and drawbacks of each. We aim to assist researchers in selecting the most suitable isolation method for their experimental requirements and questions, and to facilitate the comparison of results across different laboratories using different approaches.

## Graphical Abstract

**Graphical Abstract:**
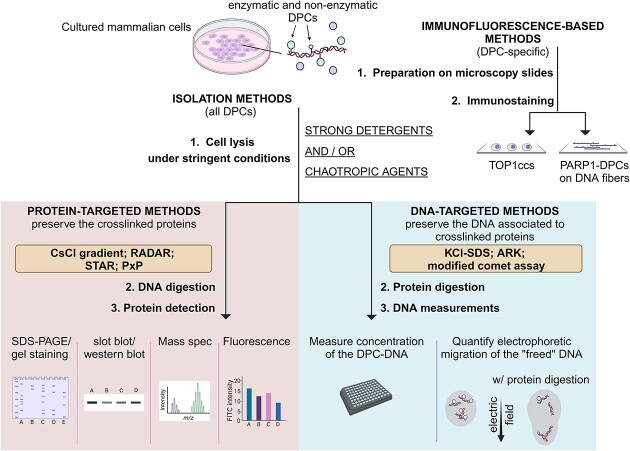

## Introduction

DNA–protein crosslinks (DPCs) are common and potentially mutagenic DNA lesions that threaten genome stability if not effectively resolved. DPCs can manifest in different forms, with some being specific and transient physiological intermediates of regular enzymatic reactions. Examples include DPCs generated by topoisomerases that relax or initiate decatenation of DNA during DNA replication and transcription (1), DPCs formed by DNA polymerase beta at oxidative lesions during DNA repair (2), and DPCs generated by HMCES at abasic sites (3). Specific, stable DPCs can arise from abortive topoisomerase reactions in cells treated with topoisomerase poisons (4). In contrast, non-specific DPCs arise from crosslink formation between DNA and a variety of proteins normally in proximity to DNA. Non-specific DPCs are induced by ionising radiation, environmental chemicals and metabolic by-products such as acetaldehyde and formaldehyde. Detailed insights into the nature and the repair mechanisms of specific and non-specific DPCs can be found elsewhere (5–15).

Since DPCs block DNA transcription, replication and repair, numerous potent therapeutic strategies have been developed aimed at exploiting DPC toxicity. Fluoroquinolone antibiotics (ciprofloxacin, levofloxacin), which poison the bacterial type II topoisomerases, are used to treat a wide range of bacterial infections. Topoisomerase poisons are used in cancer therapy to create stable DPCs with TOP2 (doxorubicin, etoposide) or TOP1 (camptothecin and its derivatives topotecan, irinotecan, belotecan, and the antibody-drug conjugate trastuzumab-deruxtecan). PARP inhibitors olaparib and talazoparib, which generate tight, DPC-like complexes between PARP-1/2 and DNA, effectively induce cell death in cancer cells (16,17). Similarly, 5-aza-2′-deoxycytidine (5-aza-dC), an anti-leukemic drug that inhibits DNMT1 DNA methyltransferase, induces the formation of DPCs containing PARP1 at sites of CpG methylation (18). Ionising radiation, the most commonly used cancer treatment, is a potent inducer of DPCs, in addition to causing DNA double-strand breaks (DSB) and single-strand breaks (SSB). Furthermore, platinum-based chemotherapeutics (oxaliplatin, cisplatin) and nitrogen mustard compounds (cyclophosphamide, chlorambucil, melphalan) can induce DNA–protein crosslinks as well as DNA-DNA crosslinks (19,20).

### Formation, chemical structure and biological identification of DPCs

**Figure 1.**
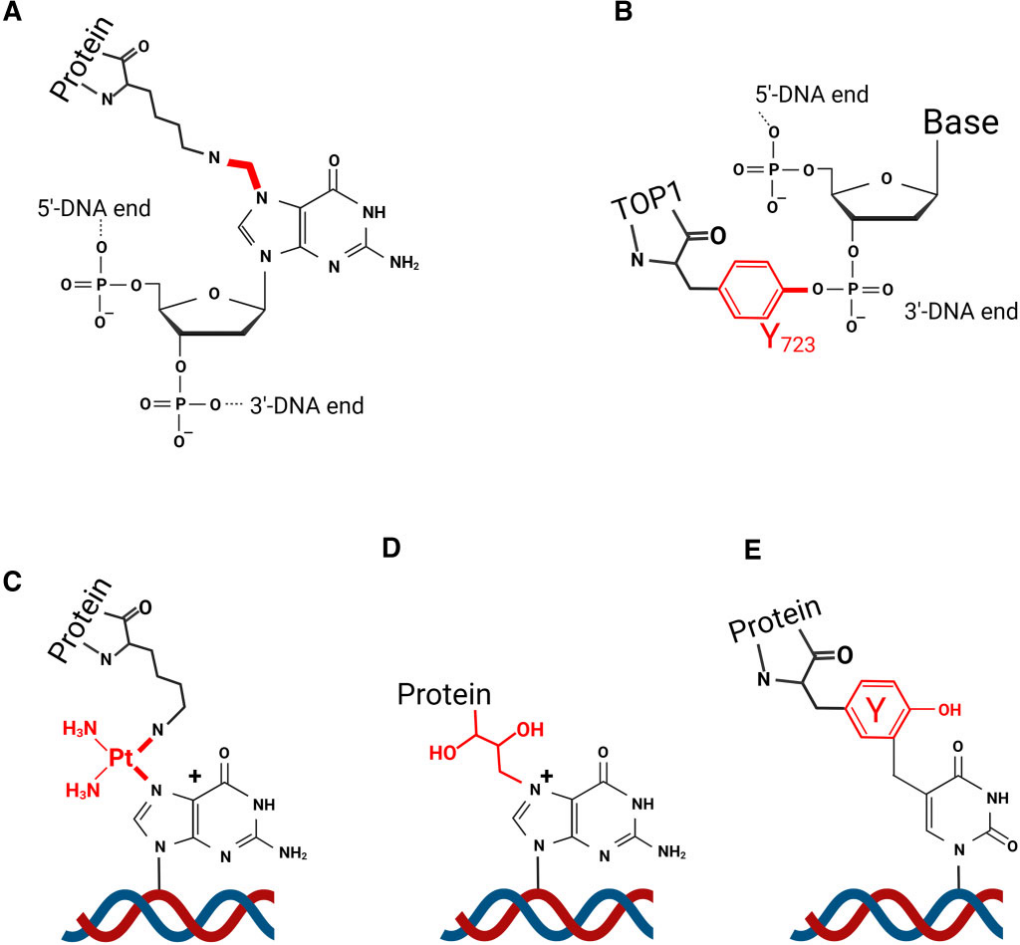
Examples of DNA–protein crosslinks, as detected *in vitro*. DPCs induced by reactions with exogenous crosslinkers (in red) or by endogenous enzyme: (**A**) chemical structure of formaldehyde-induced DPC linking a lysine residue with N7-guanine, highlighting the resulting methylene bridge. (**B**) Structure of covalent linkage of TOP1 Tyr^723^ in the TOP1 active site to the 3′-DNA end phosphate group, highlighting the Tyr bridge in red. (**C**) cisplatin-induced DPC linking a lysine residue with N7-guanine, highlighting the cisplatin bridge in red. (**D**) 1,3-Butadiene-induced DPC on N7-guanine. (**E**) Direct crosslink by a free radical between tyrosine and the methyl group in thymine.

DPCs exhibit a wide range of chemical bonds, reflecting the chemical diversity of agents that induce them and their selectivity towards specific amino acid side chains and DNA sites (Figure 1). This chemical variability has been extensively studied *in vitro* (20–26). For example, formaldehyde mediates the formation of transient *Schiff* bases with various amino acid side chains, resulting in methylene-bridge DPCs with various protein side chains (20). Platinum compounds like cisplatin preferentially target basic amino acids like arginine and lysine. Nitrogen mustards and 1,2,3,4-diepoxybutane (DEB) show a preference for cysteines (27). Not surprisingly, the N7 position on guanine is especially susceptible to DPC formation (27,28). Reactive oxygen species (ROS) and reactive nitrogen species (RNS) induce formation of DPCs between lysine and tyrosine side chains in proteins and guanine, cytosine or thymine in DNA (22,29). These diverse chemical structures confer on DPCs distinctive physicochemical properties that affect their stability and thermal lability, an important consideration when isolating and analysing DPCs.

Several research groups have made efforts to characterise the genome-wide adductome resulting from normal metabolic conditions and exposure to DPC-inducing agents (18,28,30–37). Typically, these studies involve a two-step strategy wherein DNA is initially isolated from lysed cells by a method that preserves DPCs, the DPCs are digested with proteases and the resulting peptides are subjected to the qualitative and/or quantitative compositional analysis by mass spectrometry (discussed below). The recent advancement of accurate DPC isolation methods, coupled with the considerable development of mass spectrometry-based techniques for biological samples, has provided initial insights into the DPC adductomes and has enabled the identification of proteins susceptible to crosslinking.

### Repair of DPCs

**Figure 2.**
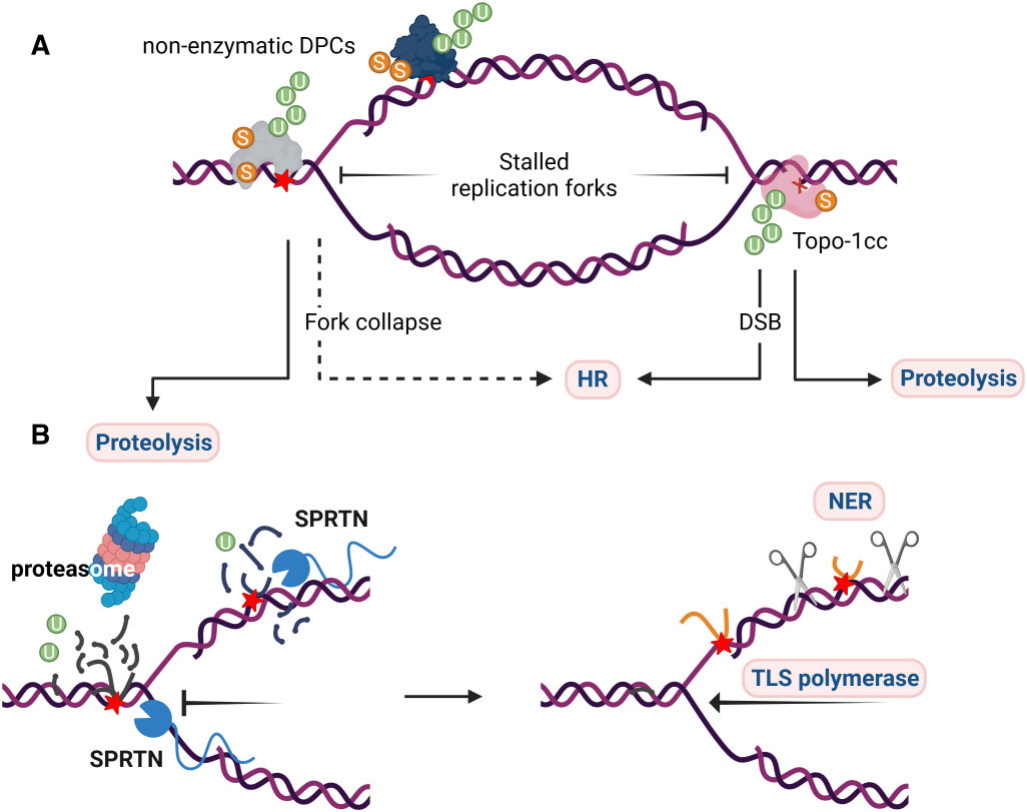
Replication-dependent and post-replicative DPC repair. The three main repair pathways are highlighted in pink boxes. (**A**) DPCs ahead of the replication fork can cause prolonged stalling and fork collapse, which is fixed by homologous recombination. Similarly, replication forks running into a TOP1cc result in single-ended DSBs which requires homologous recombination for repair. Processing of enzymatic and non-enzymatic DPCs by proteolysis is facilitated by ubiquitylation and SUMOylation. (**B**) SPRTN and the proteasome are the main proteolytic activities during and post-replication. The remnant peptide ahead of replication forks can be bypassed by a translesion (TLS) polymerase. Post-replicative remnant peptides are repaired by nucleases of the nucleotide excision repair (NER) pathway, which in mammalian cells can only operate on small DPCs.

We are now gaining a better understanding of the molecular mechanisms involved in DPC resolution. Two proteolytic repair pathways have emerged in recent years as essential mechanisms for coping with DPCs (Figure 2). During the initial steps of repair, the bulk of DPCs is cleaved by SPRTN (aka DVC1), the metazoan functional homolog of the yeast protease Wss1 (32,38–41). A second pathway relies on the 26S proteasome and is partly redundant with SPRTN for DPC proteolysis repair (33,38). Proteolysis by either SPRTN or the 26S proteasome leaves a remnant peptide attached to DNA, which can be bypassed by translesion polymerases during replication, and later excised by endonucleases or phosphodiesterases (*e.g*. TDP1/TDP2) (reviewed in (5,6,42). Canonical DNA repair pathways like nucleotide excision repair (NER) and homologous recombination (43–45) can also repair DPC, independently of or in concert with proteolytic repair (Figure 2).

Humans affected with Ruijls-Aalfs Syndrome (RJALS), also known as SPRTN (SPARTAN) syndrome, demonstrate that SPRTN is essential in humans (46,47). In this monogenic disease, biallelic hypomorphic mutations in the *SPRTN* gene result in the accumulation of DPCs and accelerated ageing and cancer (48). DPC proteolysis by SPRTN is crucial to the progression of DNA replication forks (32,49,50) and likely for translesion DNA synthesis (51–55). The role of SPRTN extends to replication-independent processes, though it remains restricted to the S- and G2- phases of the cell cycle (33,52). Functions and regulation of SPRTN have been reviewed elsewhere (5,56). Other human proteases (FAM111A, FAM111B, GCNA, DDI1, DDI2) can also repair DPCs but their roles are less defined (8,57,58) (reviewed in (59)).

DPC recognition and processing are aided by post-translational modifications on DPCs themselves (ubiquitin, SUMO and PAR) (38,57,59–63), DNA structures surrounding DPCs (64), physical and functional interactions with the p97 ATPase and with FANCJ (60,65,66), as well as by post-translational modifications on SPRTN (phosphorylation by CHK1, deubiquitylation by USP7, USP11 and VCIP135, and acetylation) (49,67–69). Regulation of DPC proteolysis repair by post-translational modifications has been reviewed in (70).

### Intention of this review

Appreciation of the importance and variety of DPCs has grown in recent years. Key to systematic investigation of DPC formation and resolution is a method that recovers covalently-bound proteins while eliminating loosely-bound proteins, and facilitates quantitative analysis of the isolated DPCs. The goal of this review is to highlight the technical advances that enable rapid, systematic and cost-effective analysis of DPCs. We hope that this will enable researchers to identify the most suitable DPC isolation method for their needs and to facilitate the comparison of results across different laboratories using different approaches.

By detecting either bound protein or bound DNA, DPC preparation methods can be classified into protein-targeted methods (discussed in **Section 1**) and DNA-targeted methods (discussed in **Section 2**). Additionally, we discuss the use of antibodies to detect specific DPCs (**Section 3**). We evaluate the advantages, limitations and applications of the most frequently used methods for isolating and/or detecting DPCs in mammalian cells (Table 1), based on published literature as well as our own practical experience. Additionally, we delve into the identification of the proteins implicated in DPCs.

**Table 1.** Summary of DPCs isolation and detection methods, their advantages and limitations. References are not exhaustive

Method group	Technique	References	Advantages	Challenges
Protein-Targeted methods	CsCl gradient	(76–84)	Well-established technique.	Laborious and time-intensive. Requires specific instruments and reagents.
	RADAR	(3,18,32,63,65,68, 85,86,96,99)	Simple execution. Sensitive and specific.	Potential contaminations with non-crosslinked proteins and RNA-protein crosslinks.
	STAR	(31)	More stringent isolation. Removal of RNA. Enrichment of proteolytic fragments.	High protein content. Unclear improvement over RADAR.
	PxP	(33)	Able to isolate large adducts. Highly confident protein identification	Potential false negatives. Lack of scalability.
Detection of DPCs isolated by protein-targeted methods	Immuno-detection	(3,18,31–33,59,65, 68,86,95,96)	Well-established	Requires prior DPC identification. Requires good primary antibodies.
	Radio / Fluorescence labelling	(44,84,104–107)	Sensitive. Accurate quantification.	Unable to detect proteins of interest. Pre-labelling may result in potential cross-reactivity.
	Mass spectrometry	(28,31–34,37)	Sensitive and quantitative. Powerful and versatile.	Requires specialised instruments.
DNA-Targeted methods	KCl-SDS precipitation	(32,50,57,69,121)	Reproducible. Small number of steps.	Precipitates free proteins: high background and limits to measure total DPCs
	ARK	(68,122,123)	Low background. Highly sensitive.	Does not isolate particular proteins.
	Comet assay	(125,126,131,132,135)	Fluorescence microscopy images. Alternative to biochemical isolation methods.	Low sensitivity. Not possible to detect a protein of interest.
Immunodetection with specific DPC antibodies	TOP1cc	(65,67,138,139,142)	Reproducible. Sensitive and specific to TOP1cc.	Potential weak staining in IF.
	PARP1 on DNA fibres	(152,155,156,158)	Low number of cells needed. Large number of DNA fibres Robust data.	Complex quantification.

For full reference records, see the main manuscript.

## Section 1. Protein-targeted methods for DPC preparation

Protein-targeted methods separate DNA from free proteins and analyse the proteins associated with the DNA. Early techniques used DNA-zol® (71–73) or phenol:chloroform/SDS (36), but more sophisticated approaches have since been developed, including caesium chloride density gradient, RADAR, STAR and PxP. In addition to describing these methods, we detail a diverse array of detection methods that aim to provide a qualitative, quantitative and sensitive assessment of the DPC content in a sample.

### Caesium chloride density gradient separation

**Figure 3.**
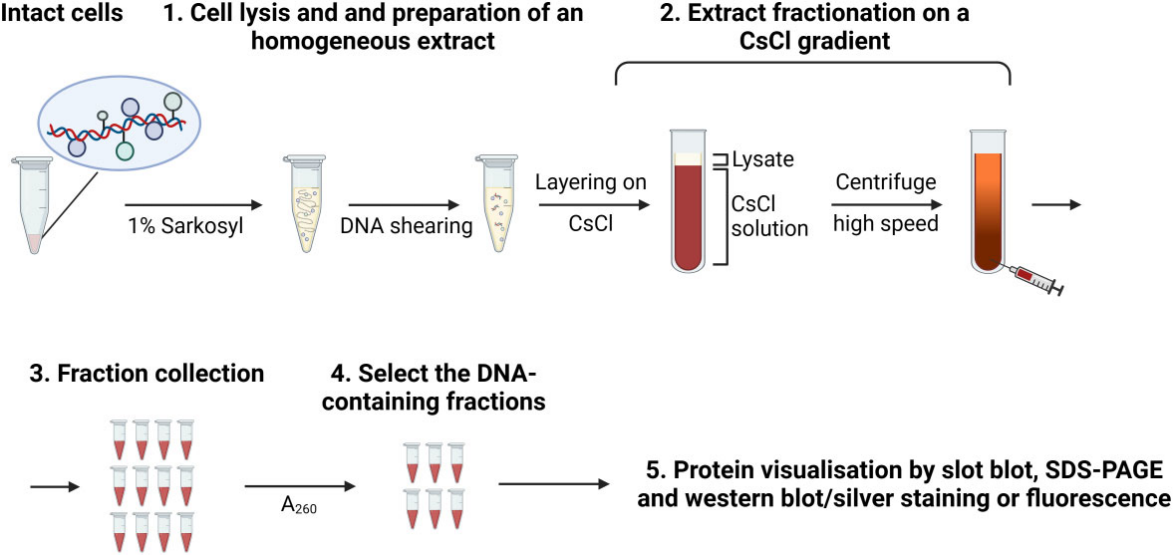
Caesium chloride gradient method. Schematic of CsCl gradient method according to (76).

One of the earliest and most versatile techniques for DPC isolation is their purification through a caesium chloride (CsCl) density gradient (74) (Figure 3). CsCl is a dense salt that creates a linear gradient during ultracentrifugation, with decreasing density from bottom to top. Cellular material of varying molecular weight but similar density aligns in the CsCl gradient upon centrifugation (75). The covalent attachment of proteins to DNA modifies its DNA density, leading to a shift in its sedimentation properties.

Briefly, cells are lysed using a high detergent buffer (*e.g*. 1% Sarkosyl) to obtain chromatin, although sequential lysis steps with milder detergents are sometimes used to enhance DNA purity, such as 0.5% NP40 followed by nuclei/chromatin centrifugation and washes with 0.5% Triton X-100 (76). DNA is sheared by passage through a syringe needle. The prepared chromatin is applied to a CsCl gradient and centrifuged until equilibrium (*e.g*. 500,000 g for 4 hours, but this depends on the size of the sample and the tube's volume). DNA-bound proteins migrate into the gradient, while free proteins remain at the top. The bottom of the tube is poked and fractions are collected. DNA-containing fractions, whether native DNA or DPCs, are commonly selected based on *A*_260_ measurement and further processed and analysed conforming to the appropriate DPC detection method.

A CsCl-based method was adapted to study DNA-bound topoisomerases and renamed ICE (In-vivo Complex of Enzyme bioassay)/ICT (In-vivo Complex Topoisomerase) assay. Originally employed to assess TOP1 association with ribosomal DNA (77), it was also applied to analyse the trapping effect of TOP1/TOP2 poisons and UV exposure (78–80). Toposomerases covalent complexes (TOPccs) are conveniently detected *via* slot blotting (2–20 μg of DNA) using a specific primary antibody (80,81). Similarly, the CsCl gradient fractions can be blotted with antibodies specific to other crosslinked proteins of interest, such as DNMT1 crosslinks induced by 5-aza-dC (82) or cytokeratins induced by the anti-tumour drug aminoflavone (83). Besides individual proteins, the CsCl isolates can be analysed for total DPCs. Isolated proteins might be post-labelled with fluorescein isothiocyanate (FITC) (see below) for subsequent analysis by fluorescence readings or western blotting against FITC. This method proved quantitative and sensitive enough to track DPC increases and repair in response to physiological doses of aldehydes (*i.e*. ≤ 220 μM) (84).

#### Advantages

CsCl gradient ultracentrifugation is a well-established method for fractionating nucleic acids, with a long history and an extensive literature.

#### Limitations

This technique has fallen out of fashion because it requires costly, legacy equipment that is rarely available in contemporary laboratories. It is also time-consuming and labour-intensive, yields preparations of variable purity and has limited scalability.

### RADAR (rapid approach to DNA adduct recovery)

**Figure 4.**
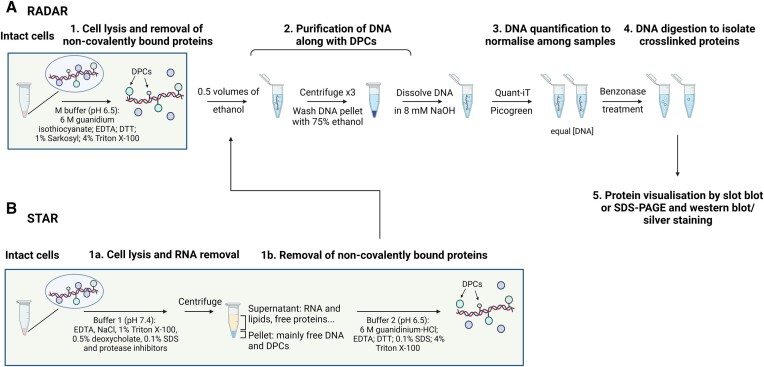
RADAR and STAR methods. (**A**) Schematic of RADAR method according to (85). (**B**) Schematic of STAR according to (31).

RADAR (rapid approach to DNA adduct recovery; Figure 4A) is currently one of the most widely used methods for the biochemical isolation of DPCs (85). It is rapid, sensitive and requires very little starting material (10^4^ cells, which yields the equivalent of 60 ng of DNA), making it suitable for high throughput analyses and ELISA immunodetection (86).

A typical RADAR extraction buffer for mammalian cells, made without proprietary reagents, contains the chaotropic agent guanidinium isothiocyanate (GTC, 6M) (85) and the detergents Triton X-100 (4%) and Sarkosyl (1%), along with 10 mM Tris-HCl (pH 6.5) and 20 mM EDTA (85). The distinctive combination of GTC with detergents creates a solution highly unfavourable to non-specific protein-DNA interactions. After extraction, nucleic acids are immediately alcohol-precipitated, the supernatant (which contains free protein) carefully removed and the pellet washed with 75% ethanol and reprecipitated. The washed pellet, which contains DNA and DPC, is resuspended in 8 mM NaOH, quantified with Quant-iT™ Picogreen™ to enable sample normalisation, and digested with benzonase to destroy nucleic acids (both DNA and RNA). The resulting protein isolate (*i.e*. the crosslinked proteins) can be analysed by various methods, including ELISA, slot-blot and Western blotting to detect specific proteins, or by mass spectroscopy for an unbiased view of the adduct repertoire. The entire adductome can be examined by silver or Flamingo staining, as specified below. Negative controls have confirmed the specificity of crosslink detection (3,87).

RADAR has been widely applied to characterise chemotherapeutically relevant DPC-inducing agents, to measure drug efficacy and to clarify the mechanism of action of anti-tumour drugs. First applied to the detection of DNMT1 crosslinks induced by the anti-tumour drug 5-aza-dC (85), RADAR has been employed to analyse the formation of TOP1 and TOP2 adducts by camptothecin (CPT) and etoposide (32,59,65,85,88,89); to compare the efficacy of conventional topoisomerase trapping agents with new topoisomerase inhibitors, such as the second generation of indenoisoquinolines, which have better pharmacokinetics and lower toxicity than CPT (90); to establish that exatecan induces TOP1cc more effectively than either CPT or topotecan (91); and to demonstrate that the compound CX-5461, originally described as an RNA polymerase I inhibitor, caused the formation of TOP2B crosslinks (92). RADAR was also used to demonstrate that lipid peroxidation by-products accumulating in CPT-treated cells are themselves capable of inducing DPC. Among these, 4-hydroxy-2-nonenal stabilises TOP1ccs with efficacy comparable to CPT, thus showing that CPT causes the covalent complex by both direct and indirect mechanisms (93).

RADAR, through its ability to co-precipitate DNA and RNA, has enabled the demonstration that TOP3B crosslinks to RNA in addition to DNA, and is the only topoisomerase known to be capable of doing so (94). RADAR has also been instrumental in characterising DPCs formed by DNA polymerase beta in response to oxidative stress (2), by histones H3/H4 in response to formaldehyde treatment (32), and by the Epstein-Barr virus EBNA1 protein (87).

The analysis of specific crosslinked substrates alongside total DPC isolates has shown a role in DPC repair for the protease SPRTN (32), the ATPase p97 and its cofactor TEX264 (65), the proteasome (3), the DNA break repair factor XRCC1 (95), the de-ubiquitylating enzyme (DUB) USP11 (68), and the exonuclease EXO1 (96) in human cells; and for the protease GCNA in *Drosophila* and zebrafish (97). Furthermore, post-translational modifications such as ubiquitylation and SUMOylation can be detected on Western blots of DPCs (59) or specific adducts (63,98), establishing a role for the ubiquitin/SUMO system in DPC repair.

Finally, RADAR isolates have been subjected to downstream mass spectrometry and Illumina sequencing (18,32,97,99). Mass spectrometry analysis of total DPCs from SPRTN-depleted cells and GCNA knock-out Drosophila embryos have defined the most abundant crosslinked proteins in these systems (32,97). In addition, it defined the relatively limited repertoire of proteins and protein families that constitute the ’adductome’ in human cells (37).

Illumina sequencing was used to sequence the regions where PARP1 covalent adducts form as a consequence of DNMT1 activity (Adduct-seq, (18)) and where TOP1ccs preferentially form (TOP1 CAD-seq, (99)).

#### Advantages

RADAR is easy to execute and relies on non-proprietary reagents and equipment commonly found in contemporary laboratories. It can be performed on human cells, tissues, *Drosophila* and zebrafish embryos, and Mycobacteria, which are notoriously difficult to lyse (85,86,97,100). RADAR is sensitive, specific, versatile and suitable for downstream applications such as mass spectrometry (32,37,97) and Illumina sequencing, provided that the DNA is resuspended in a buffer compatible with library preparation and that chromatin-immunoprecipitation (ChIP)-grade antibodies are available for the protein of interest (18,99).

#### Limitations

Insufficient removal of the non-crosslinked proteins can result in a high background. In order to minimise it, the user should pay particular attention to the following steps: (i) the cells must be lysed in the appropriate volume of buffer (advised no less than 1 ml per 2 × 10^6^ cells); (ii) following the initial ethanol precipitation, the DNA pellet must be washed carefully and extensively.

#### Useful modification

Both DNA and RNA are recovered following alcohol precipitation of cell lysates. RNA may be removed by RNase A treatment of purified nucleic acids (100 μg/ml, 30 min at 4°C) (85,86) (42,62,94).

### STAR (superior method for true DNA–protein crosslinks recovery)

The Superior method for True DNA–protein crosslinks Recovery (STAR; Figure 4B) (31) is an adaptation of the RADAR method aimed at reducing RNA-associated proteins from DPC preparations. Cells are lysed in a buffer containing detergents (1% Triton X-100, 0.1% SDS, 0.5% deoxycholate, similar to RIPA buffer) but lacking GTC. Crude nuclear pellets are recovered by high speed precipitation and centrifugation and then resuspended in a buffer containing guanidinium-HCl. Subsequently, DNA precipitation and sample treatments are carried out as per the RADAR protocol.

STAR appears sound by several criteria. It efficiently detected formaldehyde-dependent crosslinking of histone H3, an abundant DPC, in line with previous analyses (32,101). It also established that crosslinking of the RNA-dependent helicases DDX5 and DDX9 increases with increasing formaldehyde concentrations. In addition, when STAR was employed to assess DPC repair kinetics over 4 hours after treatment with 400 or 800 μM formaldehyde, the kinetics of DPC removal and the role of SPRTN in clearing DPCs within this time frame were consistent with previous studies using RADAR (32,102).

The DPC preparation by STAR was separated by SDS-PAGE and the gel was sliced into higher and lower molecular weight components (>60 kDa or <60 kDa). Proteins from each slice were eluted and analysed by mass spectrometry. The faster-migrating fraction contained peptides of proteins larger than 60 kDa in molecular weight (such as TOP1), which are presumably the result of cleavage/digestion of full-size DPCs, in line with previous reports of proteolytic repair (10,59).

Proteins isolated by the STAR method were analysed by semi-quantitative mass spectrometry and, while a repertoire of over 300 proteins was found, the majority of DPC pools contained only a few highly abundant proteins from the core histone and hnRNP families and some filamentous structural proteins. Approximately two hundred other proteins, including ubiquitin and SPRTN itself, were found to comprise a minor proportion of the DPC pool. After treatment with formaldehyde, proteins involved in RNA metabolism showed the highest increase (31).

#### Advantages

The bulk of RNA and soluble proteins are efficiently removed with the preliminary lysis step and centrifugation, which effectively only precipitates DNA and DPCs. The result is a DPC preparation devoid of cytoplasmic RNA and contamination from non-crosslinked proteins.

#### Limitations

Preliminary cell fractionation in a buffer lacking chaotropic salts may be accompanied by proteolysis and nucleolytic digestion that destroys or modifies DPCs.

#### Other considerations

As recovery of RNA is indeed intrinsic to the nucleic acid isolation, several other protocols have implemented RNase treatment to remove RNA (31,42,62,85,94). A somewhat similar step to eliminate cytoplasmic RNA was also previously implemented (18) using a proprietary detergent (MPER, ThermoFisher) to disrupt cell membranes, followed by high-speed pelleting of the nuclear material and denaturation in RADAR buffer. In this respect, the preliminary lysis for RNA removal in the STAR protocol provides limited advance.

There is no convincing evidence that the STAR protocol achieved higher levels of sample purity compared to RADAR (31). The claim appears to have been based on a comparison between a STAR preparation and a particularly contaminated RADAR preparation. For example, while DPCs isolated by RADAR have previously been shown to increase in a time- and dose-dependent manner after formaldehyde treatment, with a detectable increase at 150 μM (59), DPC accumulation in the RADAR preparation from Glumac *et al.* (31) cannot be detected even in cells treated with 400 μM formaldehyde. In addition, a comparison between the RADAR isolates and total extracts shows barely any difference in the level of cytosolic and nuclear contaminating proteins; on the other hand, histone H3, an abundant protein prone to crosslinking (32), remains undetectable in the DPC preparation. Hence, based on these contradictions with previously published reports, the benefit of cell lysis in detergent before RADAR extraction remains unclear.

### PxP: purification of crosslinked proteins

**Figure 5.**
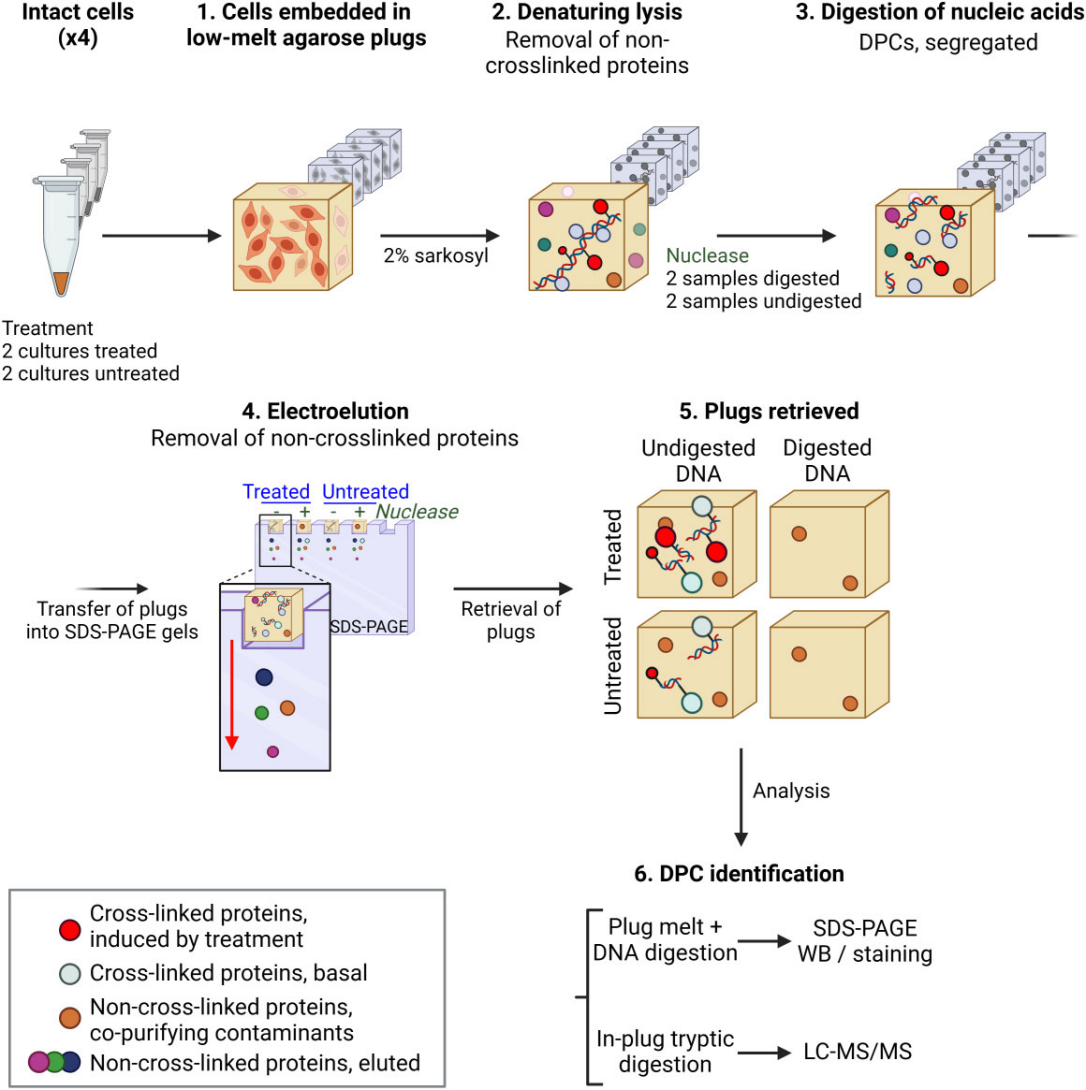
PxP method. Schematic of PxP method according to (33).

As an alternative to precipitation, DNA can be extracted from cells and immobilised on a solid matrix such as agarose, from which non-crosslinked proteins are removed by electro-elution (Figure 5). This approach to ‘Purification of x-linked proteins’ (PxP) (33) represents an adaptation of the chromosome entrapment experiments originally designed to assess interactions between prokaryotic condensin rings and DNA (103).

Briefly, 1 × 10^6^ cells per condition are harvested and embedded in 1% low melting agarose plugs and lysed in 2% sarkosyl. The plugs are then subjected to PAGE to elute the mobile cellular proteins from the agarose, while DNA and crosslinked proteins remain inside the plug due to their very high molecular weight. Plugs are washed, the agarose melted and DNA digested with benzonase to release the crosslinked proteins, for downstream analysis by western blot or mass spectrometry. For control purposes, parallel samples are processed similarly, except that DNA digestion is performed on the agarose plug before electro-elution to identify any co-purifying background contaminant proteins.

PxP followed by western blotting has been validated for the detection of TOP1cc, histone H3 and DNMT1, formed in a dose-dependent manner in response to treatment with CPT, formaldehyde and 5-aza-dC, respectively. Interestingly, this experimental strategy served to establish an additional role of SPRTN in post-replicative repair of 5-aza-dC-induced DNMT1 crosslinks. SUMOylation of the protein adduct followed by RNF4-dependent ubiquitylation initiates this replication-independent repair mechanism, generating a modified DNMT1-DPC that may be resolved by either the proteasome or SPRTN (33,61).

PxP has also been used in combination with LC-MS/MS to reveal the identity of DPCs following the exposure of HeLa cells to a high formaldehyde pulse (2 mM, 1 hour). Through label-free quantitative (LFQ) mass spectrometry, 35 proteins were significantly enriched in PxP plugs after formaldehyde exposure. Notably, five core histones (H2AC14, H2AFY, H2BC12, H3C1, H4C1) accounted for the vast majority of the formaldehyde-induced DPCs, indicating that crosslinked nucleosomes are the predominant formaldehyde-induced adducts, as expected if crosslinking is a function of protein proximity to DNA. Interestingly, the complexity of the DPC pool isolated by PxP appeared to be comparatively simpler, both in terms of number and composition, than the DPC pool isolated by alternative methods.

#### Advantages

PxP provides a high level of confidence in distinguishing DPCs from contaminants, thanks to protein identification in nuclease pre-treated control samples. By comparing the protein profiles between experimental samples and control samples, researchers can confidently discern the presence of DPCs while minimising the risk of false positives or contaminants. The degree of confidence in the results can be adjusted by establishing thresholds for the fold change increase between the experimental and control samples. For instance, by setting a threshold of a log_2_ fold increase of 2, researchers were able to confidently identify 35 proteins as formaldehyde-induced DPCs. This set included seven histones, five members of the HMG family, TOP1, TOP2A and PARP1. Consequently, PxP enhances the accuracy and reliability of DPC studies.

Notably, many proteins integral to large structures like the ribosome, proteasome, collagen, tubulin, actin and even the p97 segregase did not undergo electro-elution effectively. Nevertheless, these proteins could be readily identified as contaminants as levels were comparable in control and experimental samples.

#### Limitations

Limits on the number of cells that can be embedded in an agarose plug restricts the scalability of the method. Additionally, large protein complexes may not electro-elute from the agarose plugs. Furthermore, while the PxP procedure offers the advantage of high-confidence identification of proteins involved in DPCs, it may also present a challenge when aiming to detect all genuine DPCs. The procedure may potentially lead to false negative results. The number of proteins identified in the negative control samples pre-treated with benzonase is notably high, and less than 10% of the total identified proteins were considered to be involved in DPC formation after formaldehyde treatment. For example, this study failed to detect certain proteins that were confidently identified as formaldehyde-induced DPCs using RADAR (37). These proteins include histones H1-2, H1-3, H1-4 and H1-5, the transcription factor FACT complex subunit SPT16, the helicase DDX46, the ubiquitin-like protein SUMO2, and some ribosomal proteins. Some of these proteins were also identified as DPCs with high confidence in SPRTN-deficient cells. The discrepancies in results may be attributed to the use of different cell lines, formaldehyde doses and treatment durations. Consequently, the variety of formaldehyde-induced DPCs identified using PxP is narrower in comparison to alternative methodologies like RADAR.

### Detection of DPCs prepared by protein-targeted methods

While methods for isolating DPCs are crucial for enriching and purifying DPCs from complex biological samples, detection methods aim at identifying and quantitatively assessing the presence of DPCs in a given sample. In recent years, the development of innovative approaches has driven remarkable progress in the field of DPC detection. These advances have expanded the toolbox available for studying DPCs, incorporating principles like immunodetection and mass spectrometry, resulting in significant improvements in sensitivity and accuracy. Here, we explore various methods for detecting DPCs following a protein-targeted DPC preparation. These approaches can be protein-specific, employing immunodetection techniques such as Western blotting and ELISA, or nonspecific, utilising radio- or fluorescence labelling. Additionally, we provide an overview of the main techniques relying on mass spectrometry for the characterisation of DPCs.

### DPC detection by SDS-PAGE, western blot and ELISA

Proteins of interest within a DPC preparation isolated through a CsCl gradient, RADAR, STAR and PxP are commonly visualised by Western blot, whereby the DPC isolates are resolved by SDS-PAGE, transferred to a nitrocellulose or PVDF membrane, and probed with specific antibodies (18). The quantity of DNA required per sample depends on the cell line, the abundance of the crosslinked protein, the quality of the antibody used for detection and the desired sensitivity. In our experience with RADAR, samples containing 10–30 μg of DNA (recovered from approximately 2–6 × 10^6^ human cells) are usually adequate (18,32). Alternatively, the entire DPC preparation resolved by SDS-PAGE can be visualised using silver staining or Flamingo®, where we recommend digesting approximately 80 μg of DNA to achieve good sensitivity.

For high throughput microplate analysis, such as ELISA-based RADAR, samples containing 60 ng of DNA (10^4^ human cells) are suitable (86). Here, cells can be cultured in a multi-well plate for practicality. The inclusion of silica gel fines in the lysis buffer (4–16% v/v) facilitates DNA precipitation by centrifugation. The purified DNA is then resuspended in 8 mM NaOH, quantified, diluted to 5–500 ng/ml, and digested with benzonase. Serial dilutions of DPCs are absorbed onto an ELISA plate - taking advantage of their natural affinity for plastic in microtiter plates - and subsequently detected using a primary antibody specific for the protein of interest and an HRP-conjugated secondary antibody (86).

### Detection of DPCs by labelling

**Figure 6.**
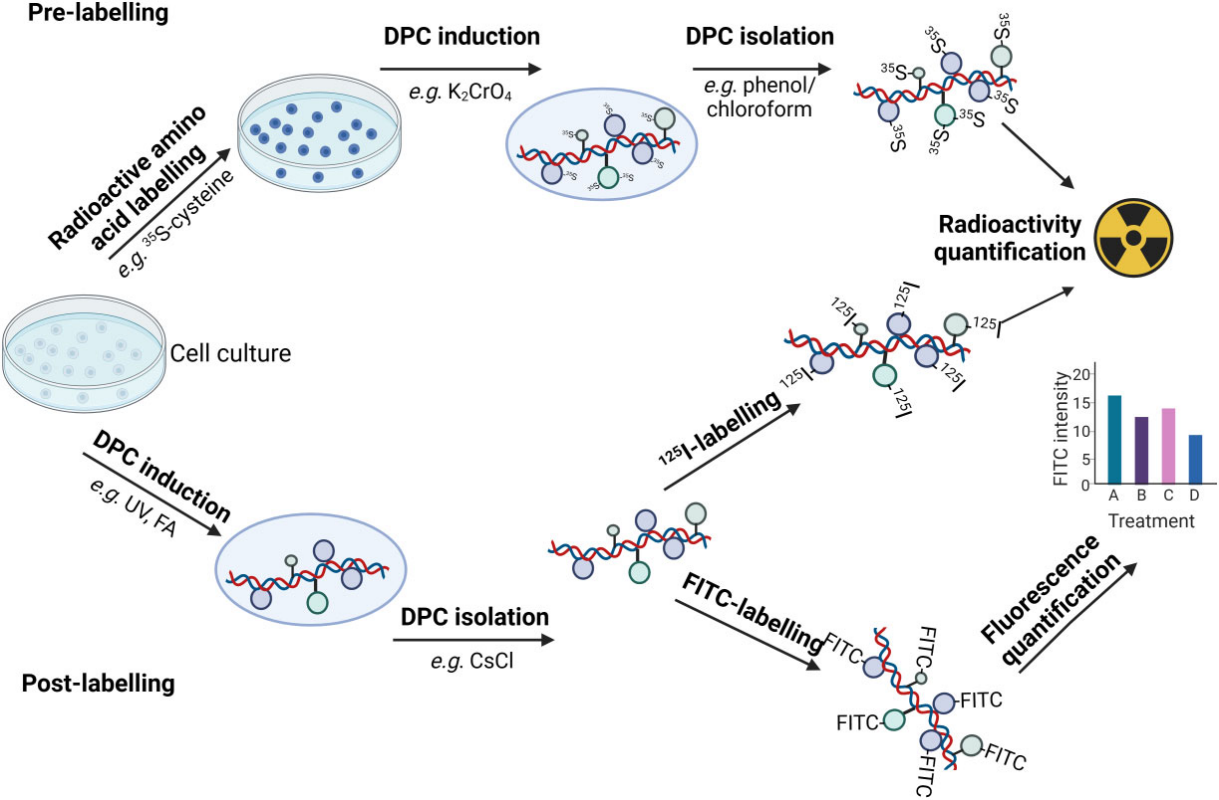
Labelling of DPCs with radioisotopic amino acids and fluorescence chemicals. Upper panel: pre-labelling method before DPC isolation according to (104). Bottom panel: post-labelling method after DPC isolation according to (44,45,84,107).

The protein component of a DPC can be labelled with radioactive isotopes or with fluorescent chemicals to facilitate its detection; this labelling can be done either in cells during cell culture (pre-labelling) or after DPC isolation (post-labelling; Figure 6).

The most common pre-labelling method involves adding a radioactive amino acid, such as ^35^S-cysteine, to the culture medium. As newly synthesised proteins incorporate the radioactive amino acid, crosslinked proteins will also be labelled and DPC frequencies can be assessed by the total radioactivity in the DPC preparation. This method was employed in Chinese hamster ovary cells (CHO) to quantify DPCs induced by chromium or nickel compounds (10–100 μM K_2_CrO_4_ or 0.2–2 mM NiCl_2_, 20 hours) (104).

Post-labelling methods most commonly employ radioactive iodine (^125^I) or fluorescein. DPC preparations can incorporate ^125^I into tyrosine residues at neutral pH in the presence of an oxidant (such as chloramine-T) (105,106). In the original study, CHO cells were treated with crosslinking agents (UV, formaldehyde, cisplatin, chromium or nickel compounds), DPCs isolated using phenol/chloroform, and proteins digested with Proteinase K. The residual DNA-bound peptides were post-labelled with ^125^I and the radioactivity of the sample was measured with a standard γ-counter, along with the 260/280 nm absorbance for normalisation against DNA (107). Other studies used fluorescein isothiocyanate (FITC) post-labelling to quantify the DPCs (purified by CsCl density gradient) from cells exposed to aldehydes and ionising radiation (44,84). The isothiocyanate group from FITC reacts with the amino groups in proteins, therefore labelling the entire DPC preparation. DPCs can be estimated by reading total fluorescence at 520 nm or by SDS-PAGE and blotting with anti-FITC antibodies. FITC post-labelling was used to analyse the involvement of the NER repair pathway in DPC repair (44). FITC labelling revealed a sensible delay in DPC repair in NER-defective cells, where conventional SDS-PAGE/gel staining did not detect any difference. With complementary experiments, the study demonstrated that only a fraction of DPCs, typically those smaller than 8 kDa, is processed by NER (44).

In conclusion, while radio-isotope labelling is not attractive due to the radioactivity involved, fluorescence-based methods are safer and should find greater use among the DPC detection methods.

#### Advantages

Although relying on conventional DPC biochemical isolation methods, the post-labelling methods are more sensitive, and quantitation can be more precise than other DPC detection systems.

#### Limitations

In these methods, all crosslinked proteins are labelled, which limits the analysis of a specific DPC. Further, the pre-labelling method does not exclude a direct reaction between radioactive amino acids and DNA, which makes it difficult to measure the real DPCs. Due to the different sensitivities of radioactive amino acids, selecting the appropriate one depends on the context of an experiment.

### Analysis of changes in DPC number and composition by mass spectrometry

**Figure 7.**
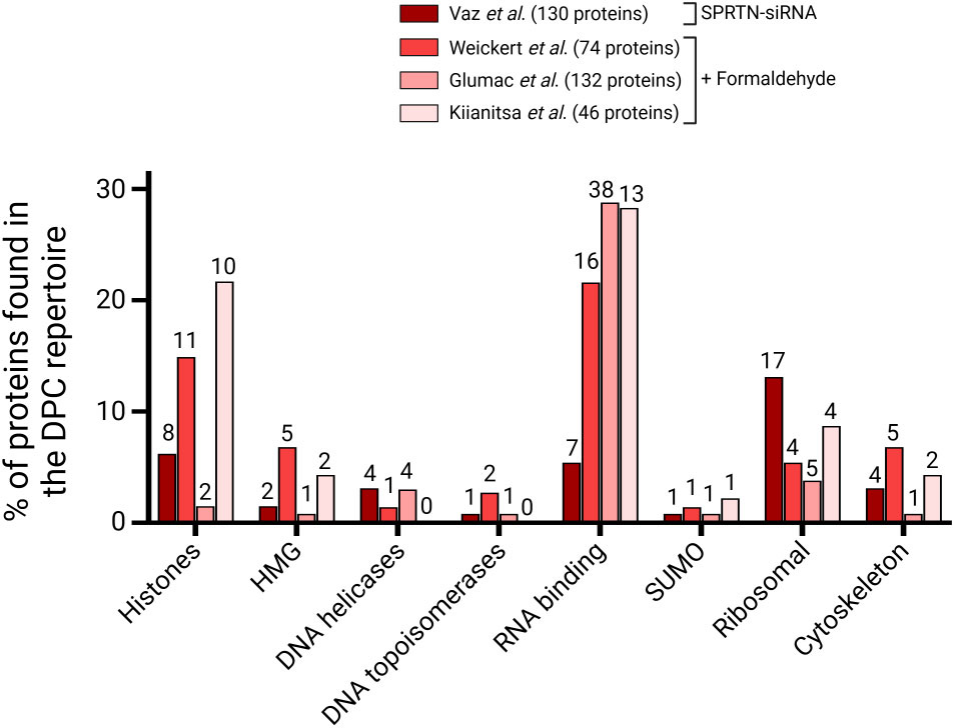
Comparison of protein repertoires identified by mass spectrometry. DPCs were induced by either depleting SPRTN from cells or by exposing cells to formaldehyde. Different DPC isolation methods: RADAR (32,85), PxP (33), STAR (31), cell types, MS instruments and bioinformatic analysis were used in each study. The graph shows the percentage of each protein group in the whole repertoire (bars) and the actual number of proteins found. HMG: high mobility group protein family.

A collective effort by several research groups to identify proteins involved in DPCs through unbiased mass spectrometry (MS) has established that DPCs are extremely complex and heterogeneous in nature. The complete repertoire of proteins found in DNA–protein conjugates could potentially encompass thousands of different proteins (Figure 7), but it has been consistently observed that the majority of DPCs are formed by only a limited set of abundant proteins that are primarily located in the nucleus and exhibit a strong affinity for DNA. DPCs at baseline levels are found in all cells, underscoring the importance of determining the frequency at which DPCs occur, to understand their relevance to genome stability.

### Absolute quantification of DPCs

The first question to characterise DPCs is to determine their occurrence rate in cells. Isotope dilution tandem mass spectrometry assay (35,36,108) is a technique capable of attaining absolute quantification of total DPC numbers in cells and tissues. In brief, the protein constituents from cellular DPCs are broken down into single amino acids and spiked with known amounts of isotopically labelled standard amino acids. Subsequent mass spectrometry analysis allows the quantification of the DPC-derived amino acids as a light-to-heavy isotope ratio. While achieving sensitive DPC quantifications, this particular technique does not provide information about the specific proteins involved. Using this technique, the number of the specific DPC dG-Me-Cys endogenously generated in rodent liver tissues was estimated at 15 DPCs per 10^8^ dG (or around 450 dG-Me-Cys per cell), above the levels presented in nasal tissue (3.6), bone marrow (2.3) and peripheral blood mononuclear cells (1.3) (108), reflecting the higher exposure of the liver to metabolic aldehydes. As expected, treatments with exogenous crosslinking agents significantly increased the frequency of DPCs. Thus, exposure of rats to airborne formaldehyde at a concentration of 15 ppm for several days increased the number of dG-Me-Cys in nasal tissue from 6.5 to 18.2 per 10^8^ dGs. Other studies estimated the number of DPCs at 200 Cys-N7G-EMA adducts per 10^8^ dGs and 6 Cys-NOR-N7G adducts per 10^8^ nucleotides in cultured human fibrosarcoma HT1080 cells treated with mechlorethamine (50 μM, 3 hours) and nornitrogen (100 μM, 3 hours), respectively (35,36). These results indicate the high frequency and formation of DPC conjugates.

### Characterisation of naturally occurring DPCs

The detection of naturally occurring DPCs can be greatly enhanced by impairing the activities of SPRTN and/or the proteasome, the two cellular mechanisms that repair them, leading to the accumulation of these crosslinks. In our efforts to identify SPRTN substrates, we isolated DPCs using RADAR from HeLa cells where SPRTN had been depleted through siRNA, as well as from parental HeLa cells (32). Subsequently, we analysed these isolates by LFQ mass spectrometry. We observed 416 different protein constituents of DPCs under basal conditions. Notably, nearly one hundred of these proteins exhibited a significant increase of more than 1.5-fold in the SPRTN-depleted samples, of which one-third had DNA-binding properties, including histones, topoisomerases and DNA helicases. Results were further validated by assays involving the expression of either SPRTN^WT^ or the catalytically inactive mutant SPRTN^E112A^. Some DPCs that did not show an increase in SPRTN-deficient cells might be resolved through SPRTN-independent mechanisms or their levels might be too low to detect changes using a mass spectrometry approach.

### Characterisation of DPCs induced by exogenous agents

Exposure to exogenous chemical crosslinking agents (aldehydes, reactive oxygen species, carcinogen metabolites, anticancer drugs or irradiation) results in significant increases in DPC number and complexity. Since formaldehyde is the main cell metabolite contributing to endogenous DPCs, treatment of cells with this agent may most accurately reproduce the protein range and chemical structures found in physiological conditions.

### DPCs induced by formaldehyde

Formaldehyde, the smallest organic chemical with crosslinking properties, is a metabolic by-product derived mainly from histone demethylation, methanol oxidation and methylamine deamination (109,110) and therefore a major source of the endogenous genotoxic challenge faced by cells (111,112). It is also a contaminant from cigarette smoke, automobile exhaust and chemical industries (113).

Kiianitsa *et al.* (37) treated CCRF-CEM lymphoblasts with 0.5, 1 and 2 mM formaldehyde for 1 hour. These doses were chosen as they moderately exceed the physiological concentration of formaldehyde in tissues and organs but are much lower than those used for extensive chromatin crosslinking in ChIP protocols (often 130 mM). Following two rounds of RADAR fractionation, including RNase A treatment, the samples were subjected to LFQ MS analysis. The result of this analysis revealed that neither the MS signal nor the number of crosslinked proteins exhibited an appreciable increase with the formaldehyde dose, confirming that massive chromatin crosslinking did not occur under these conditions. However, over 40 proteins were identified as highly reactive to formaldehyde, and their abundance in DPCs increased in a dose-dependent manner across all replicates. The majority of these DPCs were composed of various histones, HMG family members, and a diverse group of nuclear RNA-binding proteins and splicing factors. The formaldehyde-reactive DPC fraction was also highly enriched with SUMO2 peptides, a post-translational modification that targets DNA-crosslinked proteins for proteolytic repair. Notably, similar experiments in HeLa cells treated with exogenous formaldehyde at low doses (2 mM, 1 hour), followed by DPC isolation using the PxP method (33), yielded a very similar repertoire of formaldehyde-reactive proteins, predominantly composed of core histones and HMG species. They also identified other chromosome-binding proteins such as DEK, BAZ1B and HP1BP3 and the mitochondrial protein SSBP1, backing the suitability of PxP for detecting DPCs of mitochondrial origin.

Independently, Glumac *et al.* (31) undertook a qualitative MS approach to identify formaldehyde-induced (400 μM, 15 min) DPCs extracted from HeLa cells using the STAR method. Formaldehyde treatment did not significantly alter the composition of the DPC pool but enriched certain nuclear proteins that were under-represented in untreated cells (ranking within the 99% percentile of MS signal from the bottom). Among the top reactive proteins identified were some involved in ribosome function, RNA maturation, chromatin structure, DNA repair and SUMO2/3, which argues in favour of SUMOylation as a DPC repair signal (59,61,102,114).

The consistency in results across different techniques and cell types confirms the existence of a limited repertoire of proteins that exhibit high reactivity to exogenous formaldehyde at doses only slightly above the physiological level. In addition, the fact that the extent of the DPC damage may be dependent on the formaldehyde dose, as shown for histone H3, may partially explain differences across different studies.

### DPCs induced by non-enzymatic anti-tumour and contaminant agents

Chemotherapeutic crosslinkers encompass compounds like nitrogen mustards and platinum-based drugs, which can induce a wide spectrum of DNA–protein and DNA-DNA crosslinks. This field, therefore, has substantial medical significance and deserves further attention. Several studies conducted in HT1080 human fibrosarcoma cells treated with phosphoramide mustard (100 μM) or mechlorethamine (25 μM) (35), where DPCs were isolated by phenol-chloroform/SDS and analysed by qualitative mass spectrometry, identified 134 and 38 proteins, respectively, most of which were nuclear and at least one third with DNA- or RNA-binding motifs. On the other hand, treatment with cisplatin (100 μM) induced the increase of over 250 nuclear proteins, with many belonging to the HMG, histone and elongation factor families (28). Interestingly, several proteins forming DPCs were cytoplasmic, ribosomal and membrane-bound, revealing the wide range of DPC lesions induced by cisplatin. Moreover, the effect of carcinogenic environmental pollutants on DPCs has been exemplified by treating cells with 1,2,3,4-diepoxybutane (DEB, 2 mM) (34,115–117), which crosslinked 152 proteins, most of them with nucleic acid-binding motifs such as histones, HMG proteins, transcription factors, splicing factors and structural proteins.

Overall, these findings indicate that formaldehyde tends to enhance endogenous DPCs while also inducing the formation of DPCs involving proteins less prone to crosslink endogenously. The DPCs formed by nitrogen mustards, cisplatin and DEB do not differ considerably from those induced by formaldehyde. With small variations across studies, the most abundant protein components of DPCs are histones, chromatin modification/ compaction factors, transcription factors, structural proteins and a variety of ribonucleoproteins. The presence of RNA-binding proteins in the DPC pool might be attributed to the fact that many of them also possess an affinity for DNA. While contamination with RNA was not assessed in many cases, such a possibility has been ruled out before by HPLC analysis of the enzymatic digests (35,36).

## Section 2. DNA-targeted isolation methods

DNA-targeted methods estimate the amount of DPC through a quantification of DNA. The DNA engaged in DPCs is isolated from the rest either through a co-precipitation of proteins (KCl-SDS precipitation and ARK) or due to distinct electrophoretic mobility (modified comet assay). While these methods may not allow direct visualisation of crosslinked proteins, they may offer the opportunity to study the locations of DPC in the genome.

### KCl-SDS precipitation assay

**Figure 8.**
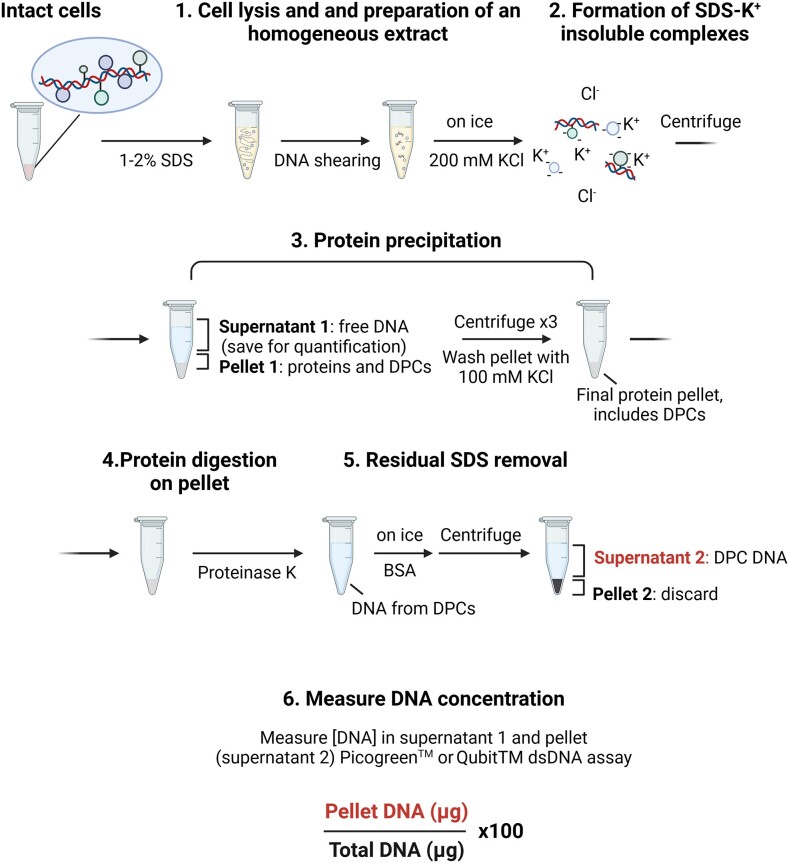
KCl-SDS precipitation. Schematic of KCl-SDS method according to (122).

This method is designed to isolate proteins, including DPCs, based on the ability of the ionic detergent SDS to bind and neutralise the cationic sites in proteins, thereby stripping weakly bound proteins from DNA (Figure 8). Subsequent addition of potassium chloride (KCl) forms an insoluble K^+^-SDS complex that is precipitated by centrifugation, leaving free DNA in the supernatant.

Initially developed for *in vitro* assays, this method was first applied to isolate avian TOP1 after reaction with ^32^P-labelled DNA. Counting ^32^P radioactivity in the precipitate quantifies the extent of TOP1 crosslinking to DNA (118). The ability of this method to isolate covalently attached proteins was substantiated *in vitro* using a mix of plasmid DNA and purified histone H1 or DNA polymerase I proteins (119). The addition of 1% SDS disrupted DNA interaction with these proteins and led to a DNA-free (unlabelled) pellet; conversely, when samples were UV-irradiated before SDS treatment, DNA was detected in the K^+^-SDS pellets, consistent with the formation of covalent bonds between DNA and histone H1 and DNA polymerase. Presence of the labelled DNA in the pellet was also observed when TOP1 was crosslinked to DNA enzymatically, in the absence of external crosslinking agents (118,119). This method was further applied to determine TOP1 and TOP2 cleavage sites on DNA (120).

The KCl-SDS precipitation method was then adapted for isolating covalently attached proteins from human and murine cells (121) and used to demonstrate defective DPC repair in human cultured cells deficient in the protease SPRTN (32,50) and the DUB USP7 (69). Typically, 1 × 10^6^ cells are lysed with 1–2% SDS. Genomic DNA is sheared by freezing/thawing cycles, sonication and/or robust vortexing. After heating at 65°C, 200 mM KCl is added on ice, forming an insoluble complex. Gentle centrifugation separates the protein pellet (from the supernatant containing free DNA), which is resuspended in 100 mM KCl and washed three times through cycles of heating, cooling and centrifugation before being resuspended in 100 mM KCl for DNA quantification. Proteins are next digested with Proteinase K, and BSA is added to quench the SDS. A final centrifugation step eliminates the SDS, yielding a supernatant containing the DPC-DNA. Concentrations of DPC-DNA (from the pellet) and free DNA (from the initial supernatant) are measured by various assays: Hoechst 33258 (121), Quant-iT™ Picogreen™ (32) or Qubit™ dsDNA HS assay (69) with standard DNA probes. The amount of DPCs is calculated as the ratio between DNA in the pellet and the total DNA (pellet plus supernatant) (named the DPC coefficient). The KCl-SDS method is sensitive enough to detect a dose-dependent increase of DPCs in response to cisplatin or formaldehyde, even at low concentrations of 75 μM formaldehyde (50,57,69,121), but, as the sensitivity depends on DNA fragment size, procedure standardisation is crucial for reproducible results.

#### Advantages

The KCl-SDS precipitation method is straightforward and can be used to analyse DPCs from various mammalian cell types. It is inexpensive, relies on standard laboratory reagents and no special lab equipment is needed. Therefore, it can be applied to a large number of samples.

#### Limitations

As this is a DNA-targeted isolation method, it cannot be used for protein visualisation or to check for a protein of interest within the DPC preparation, limiting its applications. Due to the thick and difficult-to-handle K^+^-SDS precipitate, contamination of the DPC-containing pellet by free DNA is common and generates a high background, so proper resuspension of the pellet during washing is recommended. Strong, non-covalent protein-DNA interactions that resist 1–2% SDS lysis increase the DNA read in the precipitate, contributing to background.

### ARK (advanced recovery of K-SDS precipitates) assay

**Figure 9.**
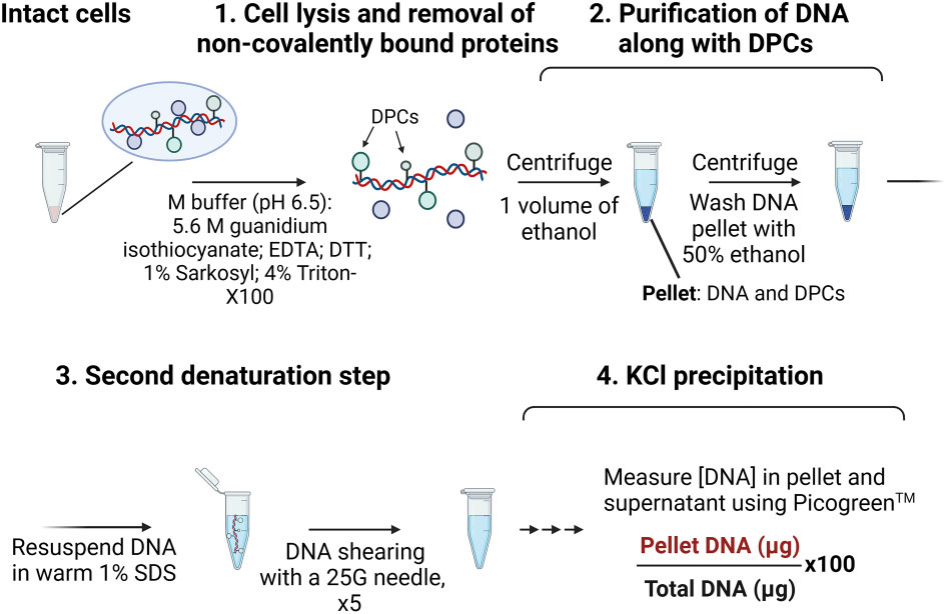
ARK assay. Schematic of the ARK method according to (121).

The ARK method (Figure 9) overcomes some limitations of the KCl-SDS assay by combining it with RADAR (122). As in RADAR, lysis with the chaotropic salt GTC in the presence of detergents releases non-covalently associated proteins and the resulting genomic DNA, including DPCs, can then be precipitated with ethanol. Performing lysis at 55°C increases stringency and reduces background. After resuspending the pellet in 1% SDS, a conventional KCl-SDS precipitation protocol is carried out. ARK, however, omits the addition of BSA to avoid interference with fluorometric readout, as DNA concentration is measured using Picogreen™. Also, as in the KCl-SDS assay, the final estimation of DPCs is determined by a ratio between the amount of protein-associated DNA and the amount of total DNA. Notably, after treating the final suspension with RNAse, no reduction in the protein background was observed, which indicates that guanidinium thiocyanate removes RNA effectively (122).

ARK’s high sensitivity was demonstrated in different cellular contexts, including HeLa, HEK293T and SK6 cells, with either *wild-type* or SPRTN, NER or Fanconi anemia-deficient backgrounds (122). Prolonged treatment with physiological doses (≤75 μM) of formaldehyde led to a significant accumulation of DPCs only when SPRTN or FANCI were depleted (∼4-fold and ∼2-fold increase after 12 hours, respectively). However, at higher doses (400 μM, 2 hours), the ARK assay showed a DPC coefficient approximately five times higher than the conventional KCl-SDS precipitation assay.

Furthermore, the ARK assay was also employed to demonstrate the formaldehyde-dependent DPC accumulation in human cultured cells lacking the DUB USP11 (68) and to detect DPCs induced by 100 mM acetaldehyde (∼2.4-fold increase) (123). As well as non-specific DPCs, the ARK assay can also be used to detect enzymatic DPCs generated by treatment with CPT and etoposide. In comparison with RADAR, ARK demonstrated similar sensitivity across various CPT doses (85,122).

#### Advantages

The protocol is fast and can be completed in 7 hours. The stringent buffer in the ARK assay effectively lowers the background compared to the lysis with SDS alone. Moreover, omitting BSA improves the Picogreen™ read sensitivity compared to the conventional KCl-SDS precipitation.

#### Limitations

Particular proteins cannot be studied using the ARK assay. However, by removing KCl-SDS from the insoluble ARK DPC precipitate with acetone (124), the protein can be solubilised for gel analysis or mass spectrometry.

### Modified comet assay

**Figure 10.**
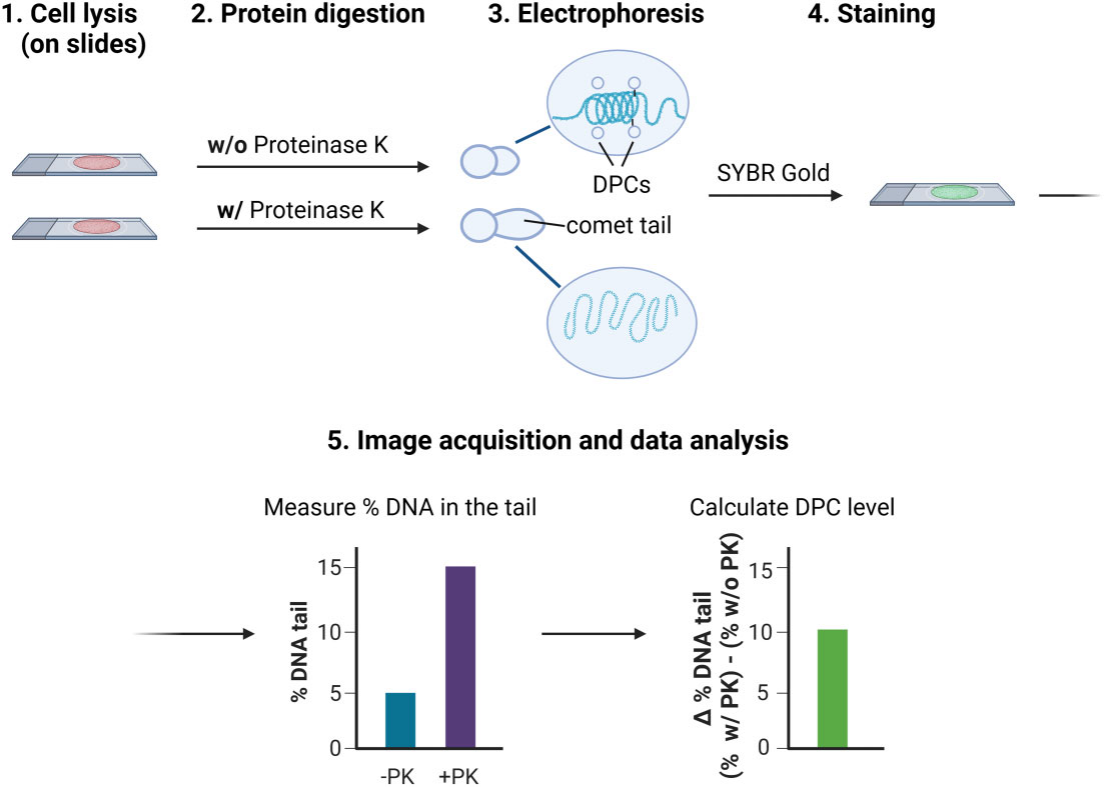
Modified comet assay. Schematic of the modified comet assay according to (131).

The comet assay is a single-cell electrophoresis-based method primarily designed for quantifying DNA breaks within individual cells. The procedure involves embedding cells in agarose on a microscope slide and subjecting them to an electric field. In this process, high molecular weight DNA remains immobile, but smaller DNA fragments (the result of DSBs) migrate towards the anode, creating a DNA comet tail (125). In alkaline conditions, the comet tail length is indicative of the overall DNA damage, including DNA DSBs and SSBs, alkali-labile sites, DNA-DNA crosslinks, and DPCs (126–130). To distinguish and specifically estimate DPCs in a sample that contains DNA–DNA crosslinks (131,132), Proteinase K is added to the slides. This enzyme digests proteins (Figure 10), enhancing the migration of DNA fragments damaged by DPCs during electrophoresis. This step is especially important for agents that induce both types of damage, such as formaldehyde.

Briefly, 5 × 10^4^ cells per condition are mixed with 0.5–1% low melting agarose and spread onto two microscopy slides per condition. The slides are soaked in a buffer containing 1% Triton X-100 and 0.5% Sarcosyl (1 hour to O/N). Proteinase K (0.8% w/v) is applied to only one of the two replicate slides. This treatment lyses the cells, unwinds DNA under alkaline conditions and digests crosslinked proteins. The slides are transferred to an electrophoresis tank filled with alkaline buffer and electrophoresis is performed for 25 min at 300 mA (25 V). Slides are allowed to air dry overnight. Then, DNA is stained with a fluorescent dye (*e.g*. SYBR gold) and visualised under a microscope. The DNA fluorescence in the comet tail (‘tail moment’) is measured using image analysis software (such as the Andor Komet) and expressed as a percentage of the total fluorescence from a single cell. The DPC levels are calculated as the difference between the tail moment with Proteinase K treatment and without it (50,131,132).

The specificity of the modified comet assay for DPCs was confirmed through a comparison of DNA damage caused by mitomycin C or cisplatin treatments, which mainly induce DNA-DNA crosslinks, and damage caused by formaldehyde (132). When Proteinase K was used, it led to an increase in the tail moment for cells treated with formaldehyde but had no such effect on the cells exposed to mitomycin C or cisplatin. Consequently, while the modified assay is less suitable for studying DNA–DNA crosslinkers (132), it has been effectively used to measure DPC levels after exposure to various common environmental contaminants, such as chromate (131), silver and titanium nanoparticles (133), and microwaves (134).

A further protocol modification enables the specific measurement of DPCs formed during the S phase (135). Cells are initially exposed to a short pulse of 5′-bromo-2′-deoxyuridine (BrdU), a thymidine analogue, to label newly synthesised DNA, before being treated with genotoxic agents. BrdU-labelled DNA is stained with an anti-BrdU primary antibody and an Alexa Fluor secondary antibody. The slides are then stained with ethidium bromide and subjected to imaging (135). This approach was employed to demonstrate that cells with a defective SPRTN protein accumulate DPCs during the S phase (50).

#### Advantages

This is the only fluorescence microscopy-based assay available to analyse non-enzymatic DPCs, alongside trapped in agarose DNA immunostaining (TARDIS) (136). It is a simple alternative to biochemical isolation methods and can complement their results.

#### Limitations

It is not possible to detect a crosslinked protein of interest using this assay, as with other DNA-targeted methods. Moreover, this assay is less sensitive than other methods, therefore it might be necessary to use a higher concentration of the crosslinking agent to detect changes (50).

## Section 3. Detection of chemotherapeutically relevant DPCs

### Immunodetection of DPCs formed by TOP1 with the TOP1cc monoclonal antibody

In order to relax supercoiled DNA, TOP1 carries out an isoenergetic reaction in which DNA cleavage is coupled with covalent bond formation between Tyr723 of TOP1 and the DNA substrate. The TOP1-DPC thus formed, also called TOP cleavage complex or TOP1cc, are of therapeutic importance because treatment with TOP1 poisons, such as CPT and related compounds, causes them to persist and promote cytotoxicity. They can be quantified by a variety of assays, including alkaline elution (137), ICE (80) and RADAR (85). However, detection was revolutionised by the development of the monoclonal antibody RRID:AB_2756354 which specifically recognises TOP1cc but not free TOP1 or DNA (138).

**Figure 11.**
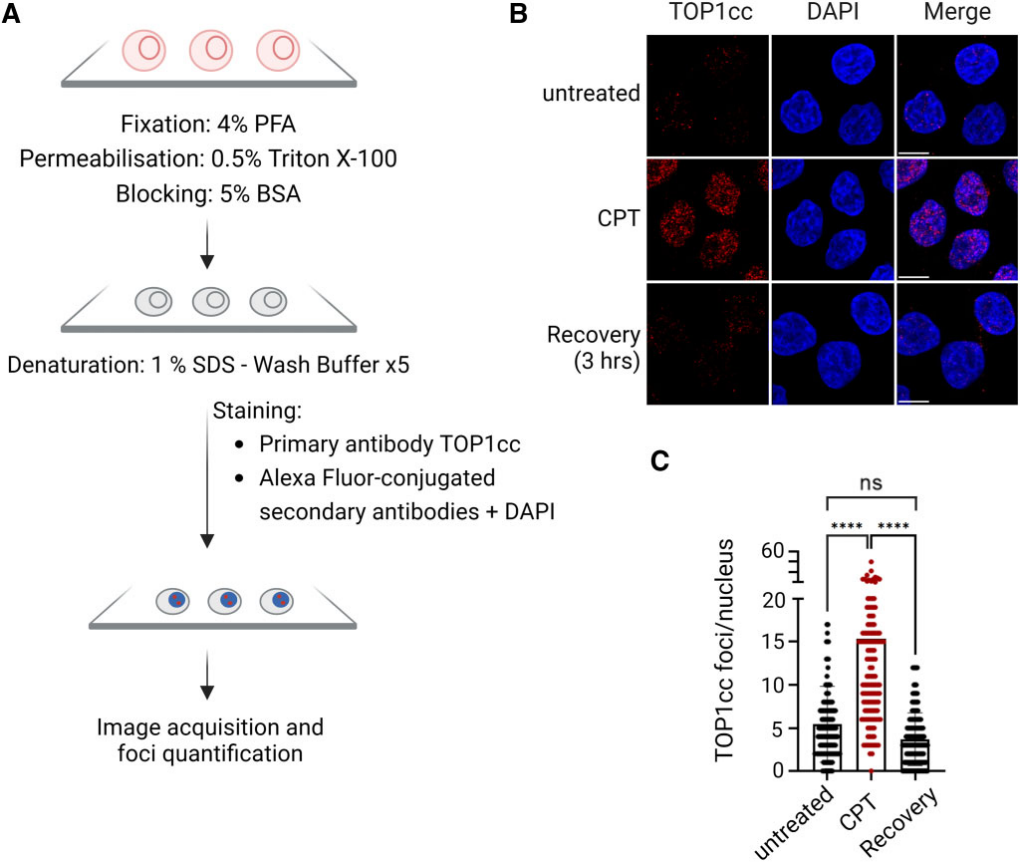
Detection of TOP1cc by immunofluorescence. (**A**) Schematic of the TOP1cc immunofluorescence protocol according to (138). (**B**) TOP1cc immunofluorescence of HeLa cells either untreated, treated with camptothecin (CPT; 50 nM, 1 hour), or 3 hours recovery after treatment. TOP1cc were stained with the primary antibody (Millipore Cat# MABE1084, RRID:AB_2756354) and the Alexa Fluor 555 secondary antibody. Scale bar, 10 μm. (**C**) Count of TOP1cc foci using Cell Profiler™. Significance determined by two-way ANOVA test. PFA: paraformaldehyde. See Supplementary material for raw data.

The TOP1cc antibody enables direct localisation and co-localisation analyses of TOP1cc without the need for cell lysis or DPC purification, and it is suitable for immunoblotting, flow cytometry and immunofluorescence. For immunofluorescence assays (Figure 11), cells are grown on coverslips, then treated, fixed, permeabilised and blocked following standard methods. To render DPCs more accessible, coverslips are further incubated in 1% SDS (5 min) and quenched with 0.1% Triton X-100. The slides are then stained with the TOP1cc antibody (1 hour, and optionally co-stained with additional primary antibodies), washed (0.1% Triton X-100) and treated with Alexa Fluor secondary antibodies. Images are acquired on a confocal microscope and TOP1cc foci or maximal mean fluorescence is quantified (see Methods).

The utilisation of the TOP1cc antibody has advanced our understanding of how TOP1-DPCs form and how they are repaired. We have learned that not only CPT and its analogues trap TOP1 on DNA, but several other genotoxic agents as well, including indenoisoquinolines, actinomycin D, and cisplatin (138). Immunofluorescence analysis has shown that TOP1cc foci colocalise with DNA damage markers, with kinetics suggesting that treatment with TOP1 poisons causes replication forks to stall, then undergo conversion to DSBs due to DNA replication fork collapse (4). Interestingly, TOP1cc foci do not co-localise with DNA damage signalling markers, suggesting that they induce secondary and distant damage on DNA (138). Immunofluorescence studies using the TOP1cc antibody have also confirmed the involvement of several key factors in TOP1cc repair, including tyrosyl DNA-phosphodiesterase 1 (TDP1) (139), the proteases SPRTN (67) and FAM111A (8), the unfoldase p97/VCP and its cofactor TEX264 (65), and confirmed that TOP1ccs are primed for proteasomal degradation by sequential SUMOylation and ubiquitylation (42,140). In addition, using the TOP1cc antibody in combination with CUT&RUN genomic sequencing has enabled the mapping of TOP1 hotspots, which were found to localise mainly in intronic regions (28.9%) and in promoters (31.6%), opening new avenues to characterise fine regulation of protein expression (141).

The TOP1cc antibody also has clinical utility. Its high sensitivity has enabled measurement of TOP1cc repair kinetics following exposure to low CPT doses (65), and has made it possible to predict the sensitivity of acute myeloid leukaemia cells to TOP1 poisons (142). The use of the TOP1cc antibody as a correlating biomarker is currently in a phase II clinical trial (*ClinicalTrials.gov NCT03289910*) (142). Furthermore, T-cells derived from individuals with chronic viral infections (hepatitis C, hepatitis B, HIV) were shown to accumulate TOP1cc foci, a finding with implications for cancer patients with these chronic infections, as the use of CPT analogues can lead to immunodeficiency (143). Additionally, endogenous TOP1ccs accumulate in Huntington's disease cells and in SPRTN hypomorphic mice liver cells, potentially exacerbating genomic instability and liver cancer, respectively (144,145).

#### Advantages

The TOP1cc monoclonal antibody is specific and does not cross-react with etoposide-stabilised TOP2-DPC (138). It is very sensitive and detects TOP1cc in untreated cells (65,139) and in cells treated at low CPT concentrations: 10–30 nM for immunoblotting, 25 nM for immunofluorescence and 16 nM for flow cytometry (138). The antibody has been used in a variety of cell lines, including A549 (138), RPE-1 (65), myeloid cells (142), rhabdomyosarcoma cells (139), MEFs (145), as well as in astrocytes derived from mouse brain (146). It can be combined with other antibodies without disturbance to co-stained proteins such as γ-H2AX (138). Using cell imaging to visualise TOP1ccs in cells provides spatial resolution that cannot be achieved by biochemical analysis alone.

#### Limitations

Intense background and non-specific staining may be exhibited by some cell types or staining conditions. More washes or the use of a different secondary antibody may improve results. For example, we noticed that, in HeLa cells, donkey Alexa Fluor 555 secondary antibody (RRID AB_2536180) produced a better signal than goat Alexa Fluor 488 (RRID AB_2534069). When growing loosely attached cells in coverslips lacking a poly-L-lysine coating, the denaturing step of SDS can be problematic.

### Visualisation of PARP1-DPC on DNA fibres

Poly(ADP-ribose) polymerase 1 (PARP1) is an enzyme that acts as a sensor of various forms of DNA damage (147,148). It catalyses poly-ADP-ribosylation of itself and other proteins to facilitate the recruitment of downstream repair factors, most notably during single-strand break repair, base excision repair (BER) and Okazaki fragment processing. PARP is a therapeutic target, and PARP inhibitors (olaparib, rucaparib, niraparib, and talazoparib) have been approved for the treatment of prostate, ovarian, breast and pancreatic cancer, targeting a synthetic lethal effect between PARP inhibition and deficiencies in homologous recombination (149,150). PARP inhibitors have been shown to induce the formation of a complex resembling a DPC, although there was some debate about whether these were true covalent adducts or tightly ‘trapped’ complexes.

PARP1-DPC were initially studied *in vitro* using radiolabelled synthesised DNA substrates, allowing detection of crosslinked PARP1 by phosphor-imaging, and these results were complemented by clear evidence for formation of PARP1-DPC during BER (151–153). PARP1-DPC have also been shown to form at regions rich in CpG dinucleotides in response to damage caused by culture in 5-aza-dC, using the RADAR-based method, Adduct-Seq (18,32,97,99). Questions were raised regarding the role of PARP inhibition in PARP1-DPC formation, particularly whether PARP inhibition enhanced PARP1 crosslinking, and whether PARP inhibition combined with methanesulfonate (MMS) had an effect greater than MMS treatment alone (152–154).

**Figure 12.**
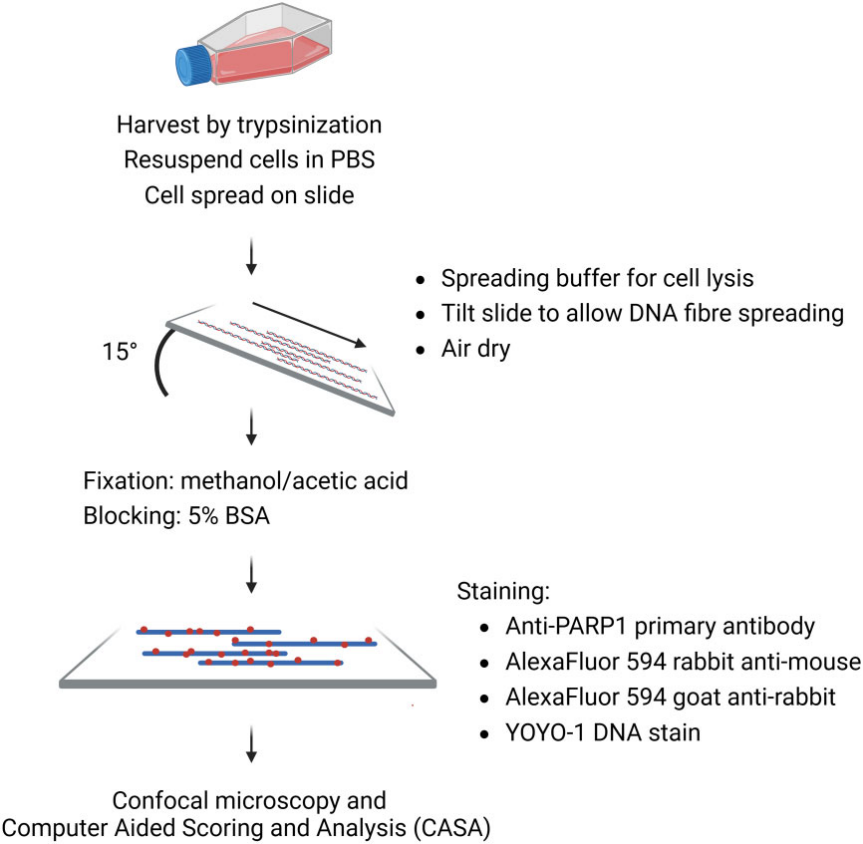
Visualisation of PARP1-DPC on DNA fibres. Schematic of the protocol as described in (152).

A technique (Figure 12) based on a classical DNA fibre spreading (155,156) incorporates PARP1 immunostaining to enable direct visualisation of PARP1-DPCs on DNA fibres (152). This technique is versatile and can provide valuable insights into how and where PARP1-DPCs are formed. After treatment, cells are harvested by trypsinisation, resuspended in ice-cold PBS, and spread onto silane-prep slides. After partial drying by evaporation, a spreading buffer containing 0.5% SDS is added to lyse the cells and gently remove non-covalently bound proteins. Slides are tilted 15° to promote the lysate to move down the slide and DNA fibres spreading. They are then air-dried before, fixed in 3:1 methanol/acetic acid, and stored at –20°C overnight. The immunostaining process begins with washing the slides in PBST buffer (0.1% Tween-20), followed by PBS, and blocking in 5% BSA. Immunostaining is carried out using an anti-PARP1 antibody (*RRID AB_394009*), followed by further washing in PBS and a second blocking step in 5% BSA. Staining is then carried out with secondary antibody (AlexaFluor 594 rabbit anti-mouse) and tertiary antibody (AlexaFluor 594 goat anti-rabbit). YOYO-1 is used to stain DNA before the slides are mounted. DNA fibres are imaged using a confocal microscope, and Computer Aided Scoring and Analysis (CASA) software (157) is used to quantify PARP1 levels per Mb of DNA. Typically, over 600 fibres are analysed per condition (152) thereby generating robust data. While conventional DNA fibre spreading techniques use thymidine analogues like 5-bromo-2′-deoxyuridine (BrdU), 5-chloro-2′-deoxyuridine (CldU) and 5-iodo-2′-deoxyuridine (IdU) to pulse-label replication sites, this requires an HCl DNA denaturation step before immunolabelling which is not necessary for the above protocol. In principle, 5-ethynyl-2′-deoxyuridine (EdU) could be incorporated into DNA and labelled with a photostable AlexaFluor dye via a click reaction. This modification would allow the visualisation of PARP1-DPCs at replication sites with minimal modification to the established protocol.

#### Advantages

Each sample requires only 2–3 × 10^3^ cells (158), several orders of magnitude less than what is necessary for biochemical analogous. Samples may be characterised at a single-cell level, with individual fibres being analysed. Visualisation of PARP1-DPCs directly on DNA fibres enables spatial characterisation, for example, to observe signal clustering. The inclusion of other markers enables assessment of PARP1 localisation at DNA damage sites, just as a similar DNA fibre approach using a fluorescently-labelled aldehyde reactive probe was used to visualise abasic sites formed during BER (159).

#### Limitations

The quantification of PARP1-DPCs using the CASA software relies on the assumption that DNA stretches uniformly, providing a direct conversion between distance and Mb of DNA. However, the described DNA spreading technique is prone to the non-uniform spreading of DNA, and frequent crossing of fibres occurs, making image acquisition and analysis more time-consuming (158). The DNA combing technique, which uses specialised equipment that stretches fibres at a constant rate of 2 kb/μm, offers more accurate DNA tract length determination (158,160,161), but it involves slower preparations and includes a Proteinase K digestion step, making it incompatible with visualising DNA–protein crosslinks. Another limitation of this technique is the absence of essential controls to demonstrate whether the detected PARP1 is covalently linked to DNA. While it has been suggested that histone core proteins are removed from fibres, leaving only covalently bound proteins (141), this assertion has not been conclusively demonstrated. Nevertheless, the technique remains valid for visualising PARP inhibitors-induced trapped PARP1 (16,162).

## Conclusion and further perspectives

The proteins that make up DPCs display a diverse range of sizes and biochemical properties, which significantly influence the outcomes when isolating DPCs using different methods. Although most protein-targeted methods consistently reveal that the majority of DPCs comprise only a few abundant proteins, the observed variations in composition and occurrence in the DPC pools can be attributed to less represented protein components. Such variability poses challenges when comparing and interpreting data obtained from different isolation methods.

The methods described here have their advantages and limitations (Table 1). Researchers must carefully consider these factors when designing experiments and interpreting results, as the method choice would ultimately depend on the specific experimental requirements. For example, when studying the total DPC pool induced by a treatment, both protein-targeted and DNA-targeted based methods are valuable. Protein-targeted methods are particularly useful when studying specific DPCs or their post-translational modifications, where various subsequent approaches facilitate targeted detection. Protein-targeted methods also enable downstream applications such as mass spectrometry or sequencing. When dealing with low-abundance proteins or low starting material, more sensitive methods should be chosen, usually relying on optimised detection systems (such as specific antibodies like anti-TOP1cc) or employing amplification techniques. Whenever possible, using two or more techniques is recommended to consolidate the results.

A common challenge across all DPC preparations is dealing with proteins that bind tightly to DNA, albeit not covalently. Lysis methods with enhanced stringency, especially the inclusion of chaotropic salts, will minimise the presence of contaminating non-crosslinked proteins, which in turn reduces the background noise and enhances the sensitivity of the assay. For example, additional lysis steps were implemented in the ARK method to improve the precipitation of the KCl-SDS technique. The visualisation of DPCs in intact cells is also challenging. Currently, cell lysis is necessary for the detection of all DPCs except TOP1, where a monoclonal has been developed specifically to detect the covalently bound protein. Visualising DPCs in intact cells provides valuable information on localisation, such as whether they cluster or migrate, and on interactions with other factors. Developing a ‘DPC reporter’ and/or identifying cellular markers for microscopy screenings (akin to γH2Ax for DSBs) would significantly advance the field. Moreover, large-scale screening methods, such as those for identifying DPC repair factors, are currently challenging and time-consuming, impeding our ability to gather comprehensive data on DPCs. Therefore, there is clear potential for the development of innovative techniques to overcome these limitations and enhance our understanding of DPCs.

## Materials and methods

### Immunodetection of DNA topoisomerase 1 cleavage complexes

HeLa cells (CCL-2) obtained from ATCC were grown on coverslips and treated for 1 hour with 50 nM CPT. Recovery was performed in growing media: DMEM containing 10% foetal bovine serum (FBS) and 5% Penicillin/Streptomycin. Cells were fixed in 4% formaldehyde in PBS for 15 min at room temperature; washed with PBS; permeabilised in 0.5% Triton X-100 in PBS for 15 min at 4 °C; and blocked in 5% BSA/PBS for 1 h at 37°C. To render the DNA–protein crosslinks more accessible to the antibody, the coverslips were incubated in 1% SDS/PBS at room temperature (RT) for 5 min, then washed five times with a wash buffer (0.1% BSA, 0.1% Triton X-100 in PBS) and twice with PBS. Incubation with TOP1cc primary antibody (Millipore Cat# MABE 1084, RRID AB_2756354) diluted at 1:250 in 2.5% BSA/PBS was performed for 1 hour at RT. Before subsequent incubation, coverslips were washed once with the wash buffer for 3 min with gentle shaking and twice with PBS. Incubation with another primary antibody can be performed at this step if needed. Coverslips were washed with PBS and incubated with secondary antibody (Thermo Fisher Scientific Cat# A-31570, RRID AB_2536180) and DAPI, both diluted at 1:500 in 2.5% BSA/PBS for 1 hour at room temperature. Coverslips were mounted onto slides using Fluoromount G (ThermoFisher). Images were taken using a Zeiss 710 LSM microscope utilising a Plan-apochromat 63x lens with a 1.4 NA and oil immersion. Images were collected sequentially to avoid any overlap between dyes whilst ensuring the same MBS filter set was maintained for all acquisitions. Images were gathered in 1024 × 1024 pixel format at approximately 55% Nyquist sampling, with full Nyquist sampling not being appropriate for this experiment. Images were acquired at 12-bit with 4× averaging being utilised to help with spurious noise within the images. Analysis was carried out using ImageJ Fiji and CellProfiler™. The pipeline used for TOP1cc quantification per cell in CellProfiler™ is provided in the source data. A Two-way ANOVA test was performed for the analysis.

## Supplementary Material

gkad1178_supplemental_file

## Data Availability

The data underlying this article are available in the article and in its online supplementary material.
