# Supplementary figures and images for: Isolation and detection of DNA–protein crosslinks in mammalian cells
Source: Nucleic Acids Res. 2023 Dec 12;52(2):525–47. doi: 10.1093/nar/gkad1178 (PMC10810220; doi:10.1093/nar/gkad1178)

Figure 11 Raw data

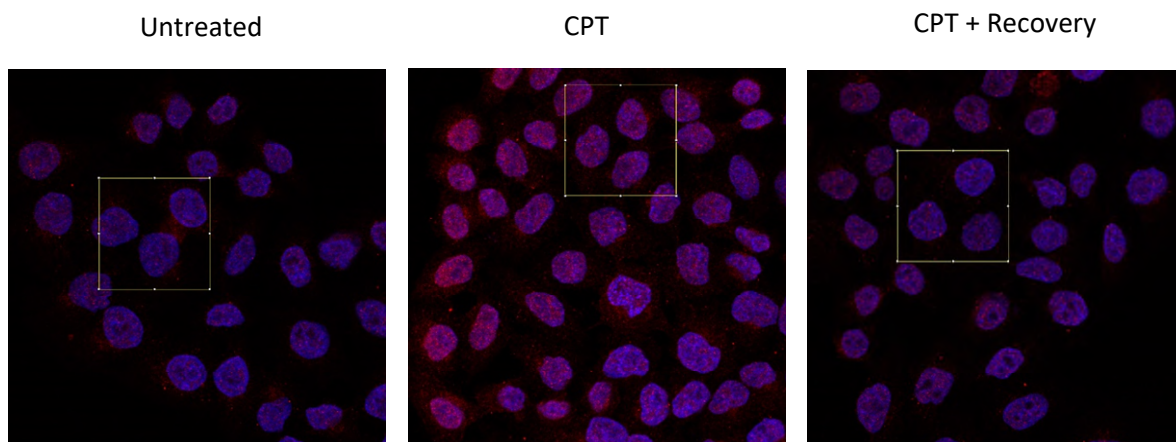

Supplement: gkad1178_supplemental_file [file gkad1178_supplemental_file.pdf]
